# *Aeromonas hydrophila* subsp. *dhakensis* Isolated from Feces, Water and Fish in Mediterranean Spain

**DOI:** 10.1264/jsme2.ME12009

**Published:** 2012-04-03

**Authors:** Consuelo Esteve, Elena Alcaide, María Dolores Blasco

**Affiliations:** 1 Departamento de Microbiología y Ecología, Universitat de València, E-46100 Burjassot, Valencia, Spain

**Keywords:** *A. hydrophila* subsp. *dhakensis*, phenotypic profile, 16S rRNA gene sequencing, emerging pathogen

## Abstract

Eight *Aeromonas hydrophila*-like arabinose-negative isolates from diverse sources (*i.e.,* river freshwater, cooling-system water pond, diseased wild European eels, and human stools) sampled in Valencia (Spain) during 2004–2005, were characterized by 16S rRNA gene sequencing and extensive biochemical testing along with reference strains of most *Aeromonas* species. These isolates and all reference strains of *A. hydrophila* subsp. *dhakensis* and *A. aquariorum* showed a 16S rRNA sequence similarity of 99.8–100%, and they all shared an identical phenotype. This matched exactly with that of *A. hydrophila* subsp. *dhakensis* since all strains displayed positive responses to the Voges-Prokauer test and to the use of dl-lactate. This is the first report of *A. hydrophila* subsp. *dhakensis* recovered from environmental samples, and further, from its original isolation in India during 1993–1994. This was accurately identified and segregated from other clinical aeromonads (*A. hydrophila* subsp. *hydrophila*, *A. caviae*, *A. veronii* biovars *veronii* and *sobria*, *A. trota*, *A. schubertii* and *A. jandaei*) by using biochemical key tests. The API 20 E profile for all strains included in *A. hydrophila* subsp. *dhakensis* was 7047125. The prevalence of this species in Spanish sources was higher for water (9.4%) than for feces (6%) or eels (1.3%). Isolates recovered as pure cultures from diseased eels were moderately virulent (LD_50_ of 3.3×10^6^ CFU fish^−1^) to challenged eels in experimental trials. They were all resistant to ticarcillin, amoxicillin-clavuranic acid, cefoxitin, and imipenem, regardless of its source. Our data point to *A. hydrophila* subsp. *dhakensis* as an emerging pathogen for humans and fish in temperate countries.

The genus *Aeromonas* (family *Aeromonadaceae*) is ubiquitous in aquatic ecosystems that include chlorinated drinking water, raw sewage, and natural waters (*i.e.*, fresh and brackish water) and free-living fish in such habitats (27). *Aeromonas* species are most commonly implicated as the causative agents of gastroenteritis in tropical countries, with sepsis a fatal complication of *Aeromonas* infectious diseases, particularly in immunocompromised patients (22). In addition, some *Aeromonas* species are one of the major causative agents of diseases in reared and wild fish (2, 13).

*Aeromonas hydrophila* subsp. *dhakensis* was originally isolated from patients with diarrhea in Dhaka, Bangladesh (India) during 1993–1994 (20, 23). Since then *A. hydrophila* subsp. *dhakensis* has not been isolated in any region of the world, but many reports have focused on its relationship with other *A. hydrophila* subspecies. Thus, depending on the housekeeping genes used (*i.e.*, 16S rRNA, *rpoB*, or *dnaJ*), *A. hydrophila* subsp. *dhakensis* exhibits high divergence from *A. hydrophila* subsp. *hydrophila* and *A. hydrophila* subsp. *ranae* (31, 32) or it does not (24, 35). In addition, it has been recently reported that type strains of the species *A. hydrophila* subsp. *dhakensis* and *A. aquariorum* clustered together in the phylogenetic tree derived from concatenated *gyrB-rpoD* sequences (31). However, the phenotypic profile of these two arabinose-negative *Aeromonas* species mostly differed (20, 30), as well as their respective genomic homology (DNA-DNA relatedness) with the type strain of the species *A. hydrophila*, which was 46% in the case of *A. aquariorum (*30) but 78 to 84% in the case of *A. hydrophila* subsp. *dhakensis* (20).

Identification of aeromonads by using biochemical schemes is difficult (1) while numerical studies have obtained quite high success showing a good correlation with genotypic identification (42). Most clinical microbiology laboratories still routinely rely on easy-to-use phenotypic methods (39); therefore, key traits for discriminating at least those *Aeromonas* species relevant in clinical sources should be well defined (25, 39). In a previous study, 215 *Aeromonas* isolates were recovered from river water and cooling systems (8), from wild European eels (13), and from human feces, collected during a 1-year period at locations in the river Xúquer floodplain, a highly urbanized and industrialized metropolitan area (753,552 inhabitants) around the city of Valencia (800,469 inhabitants). Eight out of these 215 aeromonads were presumptively identified as *A. hydrophila*, despite their negative production of acid from l-arabinose. The aim of the present study was to fully identify these *A. hydrophila*-like arabinose-negative strains, and to assess their clinical and veterinary relevance. For this purpose we used 16S rRNA gene sequencing and extensive biochemical testing, and also other assays to check for its susceptibility to antimicrobials and its virulence in fish.

## Materials and Methods

### Bacterial isolates

Strains ABF132, ABF144, and ABF145 were isolated from a wild European eel caught in the Albufera lake in El Palmar (Valencia, Spain) in November 2004 (13); strains MA17 and MA26 were isolated from two freshwater samples collected in the River Júcar in Alberique (Valencia, Spain), in October 2004 and February 2005, respectively (8); and strain MA131 was isolated from the water of a cooling-system pond in Catarroja (Valencia, Spain) in November 2005 (8). Strains 133.341 and 133.343 were isolated from stools on xylose-galactosidase plates (16) at the “Servicio de Microbiología, del Hospital Universitario La Fe” (Valencia, Spain), in September 2005. Strain CECT 4588 was originally recovered from feces of a patient with diarrhea in the Netherlands as isolate AH290 (12). This was identified as “*A. hydrophila*-like arabinose-negative” by others (42).

### PCR amplification, 16S rRNA sequencing, and phylogenetic analysis

The almost complete 16S rRNA gene sequence of strains ABF132, ABF144, ABF145, MA17, MA26, MA131, 133.341, 133.343, CECT4588, *A. hydrophila* subsp. *dhakensis* CECT5743, *A. hydrophila* subsp. *dhakensis* CECT5745, *A. aquariorum* MDC310, and *A. aquariorum* MDC317 was obtained by the Colección Española de Cultivos Tipo (CECT) service (Valencia, Spain). Bacterial genomic DNAs were extracted according to a method described previously (37). Universal primers (Invitrogen, Life Technologies) used were 616V (forward) and 699R (reverse) for a stretch around 1,000 nt close to the 5′ end (targeting positions for these primers are 8–25 and 1,099–1,113, respectively [*Escherichia coli* numbering]) (5), and P609D (forward) and P1525R (reverse) targeting positions 785–802 and 1,525–1,541, respectively (*Escherichia coli* numbering) (26). PCR mixtures were composed of 5.0 μL PCR buffer (10×), 0.75 μL MgCl_2_ (100 mM), 1.0 μL dNTPs (10 mM each), 1.0 μL each forward and reverse primers (50 μM), 0.5 μL *Taq* polymerase (6 U μL^−1^; New England Biolabs) and 5.0 μL template DNA (50 ng μL^−1^) in a total volume of 50 μL. PCR amplifications of the DNA templates were performed using a PTC-100 ThermoCycler (MJ Research). The conditions for 16S rRNA gene amplification were (i) 4 min at 94°C; (ii) 30 cycles of 1 min at 94°C, 1 min 30 s at 52°C and 2 min at 72°C; and (iii) a final elongation step of 10 min at 72°C. Amplified products were examined by agarose gel electrophoresis (1.2%) and ethidium bromide staining. Purified amplicons (Mo Bio Laboratories) were sequenced by the dideoxy method using the BigDye Terminator v. 3.0 Ready Reaction cycle sequencing kit and analyzed in an ABI PRISM 3730 sequencer (Applied Biosystems). Sequencing primers were the same as those used in the amplification reaction but diluted tenfold (5 pmol).

**Fig. 1:**
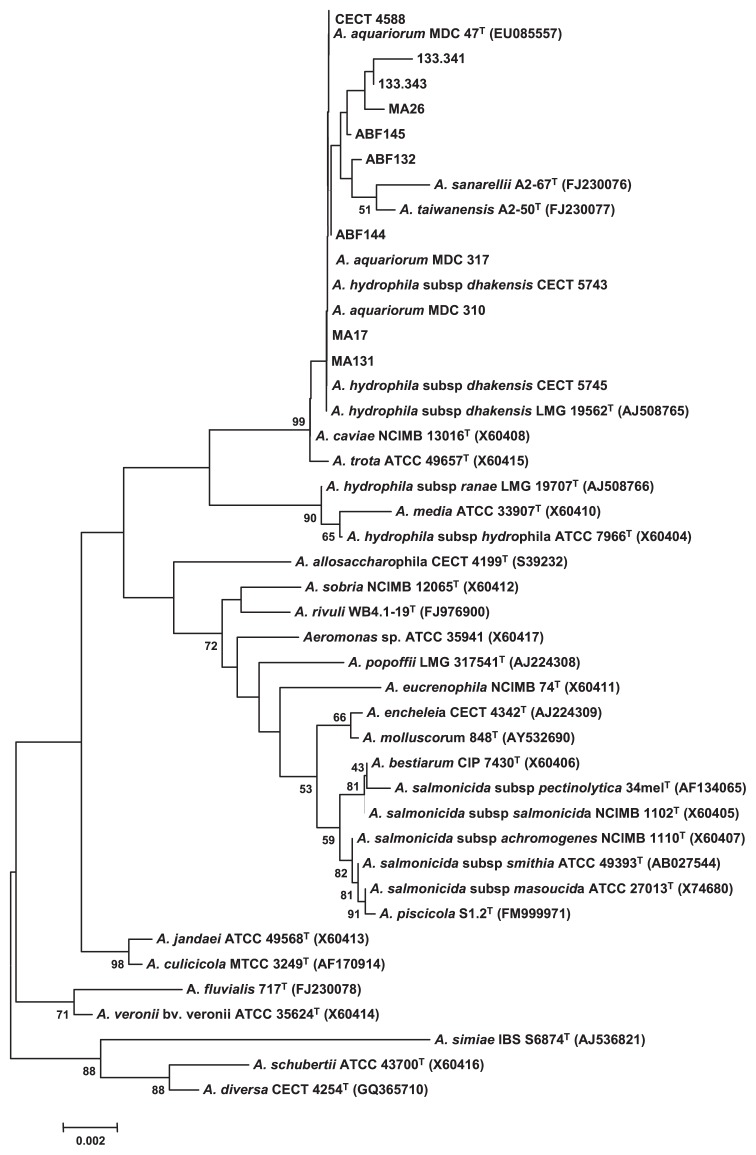
Unrooted neighbor-joining phylogenetic tree derived from the 16S rRNA gene sequences of the *Aeromonas* strains used. GenBank accession numbers for 16S rRNA gene sequences obtained in the present study are JQ034588 to JQ034600.

The sequences obtained were aligned by CLUSTAL W program, version 1.83 (41) with the sequences of the type and reference strains of the members of the genus *Aeromonas* (Fig. 1) that were available in GenBank. Genetic distances and clustering were obtained using Kimura’s 2-parameter method. Phylogenetic trees were constructed by the neighbor-joining and maximum-parsimony methods using MEGA4 program (40). Stability of the relationships was assessed by bootstrapping (1,000 replicates).

### Phenotypic characterization

Spanish isolates, strain *A. hydrophila*-like CECT4588, and reference strains of *A. hydrophila* subsp. *dhakensis* (CECT5744^T^, CECT5743, and CECT5745), *A. aquariorum* (MDC47^T^, MDC310, and MDC317), *A. hydrophila* subsp. *hydrophila* (CECT839^T^), *A. veronii* bv. *veronii* (CECT4257^T^), *A. veronii* bv. *sobria* (CECT4835), *A. sobria* (CECT4245^T^), *A. jandaei* (CECT4228^T^), *A. popoffii (*CECT4995), *A. bestiarum* (CECT4227^T^), *A. allosaccharophila (*CECT4199^T^), *A. eucrenophila* (CECT4224^T^), *A. encheleia (*CECT4342^T^), *A. trota* (CECT4255^T^), *A. enteropelogenes (*CECT4487^T^), *A. caviae* (CECT838^T^), *A. media* (CECT4232^T^), *A. schubertii* (CECT4240^T^), and *A. diversa* (CECT4254^T^) were examined in 45 tests described by us as valuable traits for identifying *Aeromonas* (42). In addition, testing of the use of urocanic acid and β-hemolytic activity against sheep blood was performed as reported by others (18, 23). All tests were incubated at 28°C. These data for *A. hydrophila* subsp. *ranae* LMG19707^T^, *A. fluvialis* CECT 7401^T^, *A. simiae* CIP 107798^T^, *A. tecta* CECT 7082^T^, *A. molluscorum* CECT 5864^T^, *A. bivalvium* CECT 7113^T^, *A. piscicola* CECT 7443^T^, *A. taiwanensis* CECT7403^T^, *A. sanarellii* CECT7402^T^, and *A. rivuli* CECT 7518^T^ were searched in published reports (3, 4, 7, 11, 15, 19, 21, 32, 33). Phenotypes were compared using the Simple Matching and Jacard’s similarity coefficients, and clustering was achieved by the unweighted pair group mathematical averaging method (UPGMA). All analyses were performed using NTSYSpc version 2.0.

In addition, API 20 E Strips (BioMérieux) were used in all isolates in order to know their API 20 E code.

### Antimicrobial susceptibility assays

Minimal inhibitory concentrations (MICs) of kanamycin (K), tetracycline (TET), nalidixic (NA), oxolinic (OA) acid, flumequine (UB), erythromycin (ERY), rifampicin (RD) and chloramphenicol (CHL) were determined by the microbroth dilution method (9). In addition, MICs of amoxicillin/clavulanic (AMC), cefotaxime (CTX), cefoxitin (FOX), ceftazidime (CAZ), imipenem (IPM), piperacillin (PRL), ticarcillin (TIC), aztreonan (ATM), cefepime (FEP), ciprofloxacin (CIP), netilmicin (NET), norfloxacin (NOR) and levofloxacin (LEF) were determined using E-strips (MICE; Oxoid, Madrid, Spain). Finally, susceptibility to the antibiotics tested was evaluated on the basis of MIC values obtained, in accordance with the breakpoints recommended by the Clinical and Laboratory Standards Institute (9).

### Virulence for European eel

For the challenge experiment, six young eels of around 10–20 g were challenged by intraperitoneal injection with each of the bacterial doses (10^8^, 10^7^, 10^6^, 10^5^, 10^4^, 10^3^, 10^2^, 10^1^ CFU mL^−1^), or with 0.1 mL PBS (controls). Each set of six eels was kept in a 20 L aquarium under the following conditions: i) dechlorinated tap water, ii) water temperature around 20°C, iii) oxygen concentration in water was above 90% saturation, and iv) fish were not fed. Mortality was recorded daily for 10 days and was only considered if the challenged bacterium was recovered as pure culture from the internal organs.

The bacterial strains used were ABF132, ABF144, and ABF145, which had been isolated by Esteve and Alcaide (13) from a specimen of wild European eel that suffered from hemorrhagic disease. The primary culture obtained originally from the kidney and liver of this specimen had been pure (*i.e.*, strains ABF132 and ABF144, respectively), but that recovered from the ulcer was not (13). In the latter case we found three types of colonies and, among them, the colony matching ABF154 was the most abundant (personal communication). Bacterial virulence (LD_50_ expressed as inoculated colony forming units, CFU fish^−1^) was calculated (38).

### Accession numbers for the 16S rRNA sequence data obtained from GenBank

EU085557 (*A. aquariorum* MDC 47^T^), FJ230076 (*A. sanarellii* A2-67^T^), FJ230077 (*A. taiwanensis* A2-50^T^), AJ508765 (*A. hydrophila* subsp. *dhakensis* LMG 19562^T^), X60408 (*A. caviae* NCIMB 13016^T^), X60415 (*A. trota* ATCC 49657^T^), AJ508766 (*A. hydrophila* subsp. *ranae* LMG 19707^T^), X60410 (*A. media* ATCC 33907^T^), X60404 (*A. hydrophila* subsp. *hydrophila* ATCC 7966^T^), S39232 (*A. allosaccharophila* CECT 4199^T^), X60412 (*A. sobria* NCIMB 12065^T^), FJ976900 (*A. rivuli* WB4.1-19^T^), X60417 (*Aeromonas* sp. ATCC 35941^T^), AJ224308 (*A. popoffii* LMG 317541^T^), X60411 (*A. eucrenophila* NCIMB 74^T^), AJ224309 (*A. encheleia* CECT 4342^T^), AY532690 (*A. molluscorum* 848^T^), X60406 (*A. bestiarum* CIP 7430^T^), AF134065 (*A. salmonicida* subsp. *peptinolytica* 34mel^T^), X60405 (*A. salmonicida* subsp. *salmonicida* NCIMB 1102^T^), X60407 (*A. salmonicida* subsp. *achromogenes* NCIMB 1110^T^), AB027544 (*A. salmonicida* subsp. *smithia* ATCC 49393^T^), X74680 (*A. salmonicida* subsp. *masoucida* ATCC 27013^T^), FM999971 (*A. piscicola* S1.2^T^), X60413 (*A. jandaei* ATCC 49568^T^), AF170914 (*A. culicicola* MTCC 3248^T^), FJ230078 (*A. fluvialis* 717^T^), X60414 (*A. veronii* bv. *veronii* ATCC 35624^T^), AJ536821 (*A. simiae* IBS S6874^T^), X60416 (*A. schubertii* ATCC 43700^T^), GQ365710 (*A. diversa* CECT 4254^T^).

### Nucleotide sequence accession numbers

GenBank accession numbers for 16S rRNA gene sequences obtained in the present study are JQ034588 to JQ034600.

## Results and Discussion

### Phylogenetic analysis

The 16S rRNA gene sequence was obtained from eight *A. hydrophila*-like arabinose-negative isolates, from strain CECT4588, and from *A. hydrophila* subsp. *dhakensis* CECT 5743 and CECT 5745, and *A. aquariorum* MDC310 and MDC317 (GenBank accession numbers: JQ034588 to JQ034600). These were compared with those of *A. hydrophila* subsp. *dhakensis* LMG 19562^T^ and *A. aquariorum* MDC 47^T^ and with those from other *Aeromonas*-type strains (Fig. 1). The 16S rRNA gene sequence similarity among the *A. hydrophila*-like arabinose-negative Spanish isolates, strain CECT 4588, and the *A. hydrophila* subsp. *dhakensis* and *A. aquariorum* reference strains was 100–99.8% (0 to 3 bp differences), in accordance with that reported solely for the type strain of these species (31). These sequence similarity values of the 16S rRNA gene were in line with those reported, among strains, for other *Aeromonas* species (7, 10, 15, 19, 28, 29, 32, 33). Thus, our phylogenetic results indicated that *A. hydrophila* subsp. *dhakensis* and *A. aquariorum* strains are extremely similar, suggesting that their genomic homology should be checked, especially as experiments of DNA-DNA hybridization between them fail to be performed.

**Fig. 2:**
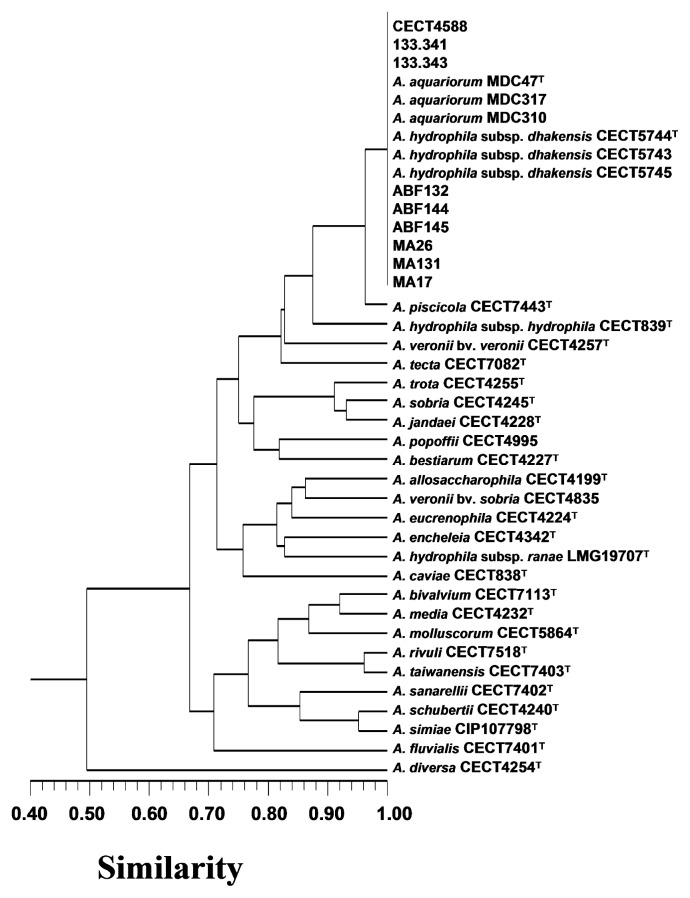
Phenogram obtained from numerical analysis of 48 phenotypic test results using the simple matching coefficient (S_SM_) and unweighted pair group method with arithmetic averages (UPGMA).

On the other hand, *A. hydrophila*-like arabinose-negative Spanish isolates, strain CECT 4588, and the *A. hydrophila* subsp. *dhakensis* and *A. aquariorum* reference strains belonged to the “*A. caviae-A. trota*” branch (Fig. 1) of the *Aeromonas* phylogenetic tree, which also includes the species *A. sanarelli* and *A. taiwanensis* (4). At present, 16S rRNA gene sequencing is widely available in reference laboratories, while biochemical testing is still appropriate for separating those *Aeromonas* species which are phylogenetically close (17). Regarding this finding, the fifteen strains tested by us were clearly segregated from the species *A. caviae* and *A. trota* (Table 1; Fig. 2), as well as from *A. sanarelli* and *A. taiwanensis* (Fig. 2)

### Phenotypic analysis

The *A. hydrophila*-like arabinose-negative isolates, strain *A. hydrophila*-like CECT 4588, and type and reference strains of the species *A. hydrophila* subsp. *dhakensis* and *A. aquariorum* were grouped by numerical analysis at 100% phenotypic similarity (S), using both simple matching (S_SM_) (Fig. 2) and Jaccard’s (S_J_) coefficients. Moreover, they were all clearly segregated from the other type strains of the *Aeromonas* species whose phenotypic profile was included in the numerical analysis (Fig. 2). All strains included in the “*A. hydrophila* subsp. *dhakensis-A. aquariorum*” cluster (Fig. 2) showed the key responses detailed in Table 1. In addition, they all were positive for: motility; cytochrome-oxidase; catalase; gluconate oxidation; O/F metabolism; growth with 0–3% (w/v) NaCl; production of H_2_S from cysteine; production of indole from tryptophan; hydrolysis of arbutin, casein, starch, elastin, and gelatin (liquefaction); β-haemolysis of sheep red blood cells; and acid production from d-fructose, d-galactose, maltose, d-mannitol, d-mannose, sucrose, and d-trehalose; however, they all were negative for: Gram staining; production of brown diffusible pigment; susceptibility to O/129 (150 μg) (Oxoid discs); growth with 6% (w/v) NaCl; hydrolysis of urea; and acid production from d-amygdalin, dulcitol, d-fucose, *meso*-inositol, melibiose, d-raffinose, d-sorbitol, and d-xylose.

Phenotypic profile of the “*A. hydrophila* subsp. *dhakensis-A. aquariorum*” cluster exactly matched that described for *A. hydrophila* subsp. *dhakensis* (20) but differed from that described for *A. aquariorum* (30). Previous data on the response of *A. aquariorum* strains to the VP test are confusing; the species was originally described as VP-negative (*i.e.*, in text) and -positive (*i.e.*, in the diagnostic table) simultaneously (30) and hence were reported as VP-positive (7, 34) or VP-negative (4, 15) in subsequent reports. Once again, species *A. aquariorum* was originally described as both negative (*i.e.*, in the diagnostic table) and positive (*i.e.*, in text) for the utilization of dl-lactate (30), but was later reported as dl-lactate-negative by taxonomic reports (7, 15, 34), mostly based on the diagnostic table (30). We have found that the three *A. aquariorum* strains assayed by us displayed a clear positive response in the VP semisolid medium (42) and in the Lactate utilization medium (18) respectively, similarly to that shown by Spanish isolates as well as by the reference strains of *A. hydrophila* subsp. *dhakensis* (Table 1). It should be noted that our results came from the first extensive phenotypic characterization that compares strains of *A. aquariorum* and of *A. hydrophila* subsp. *dhakensis*, along with other *Aeromonas* species. Accordingly, other authors have recently reported the positive responses to both, VP test and dl-lactate utilization, for *A. aquariorum* strains isolated from diverse sources in Western Australia (6).

In line with the present results, *A. aquariorum* cannot be differentiated from *A. hydrophila* subsp. *hydrophila* by the use of dl-lactate, contrary to other reports (14). In fact, the pair *A. hydrophila* subsp. *dhakensis*-*A. aquariorum*, which shared an identical phenotype, was separated in our study from *A. hydrophila* subsp. *hydrophila* by its inability to produce acid from l-arabinose and l-fucose, and its use of urocanic acid, and further by its ability to hydrolyze sodium dodecyl sulfate (SDS) (*i.e.*, alkyl sulfatase activity) (Table 1). Finally, we have identified the eight *A. hydrophila*-like arabinose-negative Spanish isolates, and strain CECT4588, as belonging to the species *A. hydrophila* subsp. *dhakensis*, on the basis of the overall results (Table 1; Figs. 1 and 2). The API 20 E profile for all strains included in the “*A. hydrophila* subsp. *dhakensis-A. aquariorum*” cluster was 7047125, which previously had shown very little prevalence (*i.e.*, 9%; 8 out of 86), which was obtained from *Aeromonas* strains (13). In addition, we found that *A. hydrophila* subsp. *dhakensis* can be accurately identified and segregated from other clinical aeromonads, such as *A. hydrophila* subsp. *hydrophila*, *A. caviae*, *A. veronii* biovars *veronii* and *sobria*, *A. trota*, *A. schubertii* and *A. jandaei* (25, 39), using biochemical key tests (Table 1).

### Incidence and clinical and veterinary relevance of *Aeromonas hydrophila* subsp. *dhakensis* in Mediterranean Spain

*Aeromonas hydrophila* subsp. *dhakensis* was previously isolated in India (23), and was encountered in Valencia (Mediterranean Spain) during a 1-year study (2004–2005) in which 32 water samples (8), 75 wild European eels (13), and an unknown number of feces from humans suffering acute gastroenteritis were analyzed. Among them, the number of *Aeromonas*-positive samples was 17 (53.1%) and 20 (26.7%), respectively, with 32 patients suffering from *Aeromonas* gastroenteritis. The overall prevalence of *A. hydrophila* subsp. *dhakensis* in these *Aeromonas*-positive specimens was of 8.7% (6/69), although it was higher for water (17.7%) than for feces (6.25%) or eels (5%). Thus, we have found a wider distribution of *A. hydrophila* subsp. *dhakensis* in Spain in comparison with its unique previous finding in association with patients but not with water or fish (23). It is important to note that such water sources were agricultural ponds linked to the Xúquer river basin, which are used for the irrigation of agricultural products; the counts of *Aeromonas* in these waters ranged from (10^2^ to 10^4^ CFU mL^−1^) in winter to (10^4^ to 10^7^ CFU mL^−1^) in summer (8). In addition, wild European eels are caught in Albufera Lake for commercial purposes and are used for human consumption (13). Hence, these findings could have public health implications because *Aeromonas* infections might be transmitted through the ingestion of contaminated water or food (*i.e.*, vegetables, fish and shellfish, *etc.*), or by contact with them (22).

Our clinical *A. hydrophila* subsp. *dhakensis* isolates (*i.e.*, 133.341 and 133.343) were recovered from two siblings, a 1-year-old boy and a 6-year-old girl, who had acute gastroenteritis accompanied by bloody stools and high-grade (≥39°C) fever and required antibiotic treatment. The fact that these clinical isolates were rather multi-resistant to antibiotics (Table 2) complicated the management of this *Aeromonas*-mediated diarrhea, similarly to in other geographical areas (39). Thus, strains 133.341 and 133.343 showed the highest MICs for ticarcillin, piperacillin, amoxicillin/clavuranic, cefoxitin, imipenem, flumequine, nalidix acid, oxolinic acid, and erythromycin (Table 2); however, resistance to ticarcillin, amoxicillin-clavuranic acid, cefoxitin, and imipenem were common in the Spanish isolates of *A. hydrophila* subsp. *dhakensis* from any source (*i.e.*, stool, water, fish) (Table 2). Fortunately, the latest generation cephalosporins and the fluoroquinolones had the best inhibitory activity *in vitro* against *A. hydrophila* subsp. *dhakensis* isolates (Table 2), as also was described for other clinical *Aeromonas* (43).

The three isolates of *A. hydrophila* subsp. *dhakensis* (*i.e.*, ABF132, ABF144, and ABF145) which have been recovered from a wild European eel suffering from hemorrhagic septicemia (13), displayed an LD_50_ dose of 2.6×10^5^ to 3.3×10^6^ CFU fish^−1^ in experimental challenges using healthy eels. These data are the first report on the active role of *A. hydrophila* subsp. *dhakensis* as fish pathogen, although others have published that a clinical isolate of this species was moderately virulent for challenged trout (36). Interestingly, *A. aquariorum*, its closest species, was originally recovered from imported ornamental fish that showed symptoms of weakness (30).

In summary, species *A. hydrophila* subsp. *dhakensis* can be recovered from natural waters, fish, and clinical specimens in Mediterranean Spain. This result suggests its potential role as a waterborne pathogen for humans and fish in temperate countries. In fact, strain CECT4588 was recovered from feces of a patient with diarrhea in the Netherlands in the eighties. *Aeromonas hydrophila* subsp. *dhakensis* can be identified by biochemical key tests. In the present study the phenotypic profile of *A. hydrophila* subsp. *dhakensis* was also demonstrated by the type and reference strains of *A. aquariorum*. Up to now, species *A. hydrophila* subsp. *dhakensis* as well as its closest species *A. aquariorum* have been reported to be distributed throughout warm countries (6, 14. 23, 30). The present results constitute the first report of *A. hydrophila* subsp. *dhakensis* from a temperate country, suggesting the worldwide distribution of this species.

## Figures and Tables

**Table 1:** Key phenotypic profile of the *A. hydrophila* subsp. *dhakensis*–*A. aquariorum* cluster^a^ compared with *Aeromonas* type strains characterized in the study

Biochemical test	*A. hydrophila* subsp. *dhakensis*–*A. aquariorum* cluster	*A. hydrophila* subsp. *hydrophila*	*A. caviae*	*A. veronii* bv. *veronii*	*A. bestiarum*	*A. encheleia*	*A. eucrenophila*	*A. media*	*A. veronii* bv. *sobria*	*A. trota*	*A. popoffii*	*A. sobria*	*A. jandaei*	*A. allosaccharophila*	*A. schubertii*	*A. diversa*
Hydrolysis of arbutin	+	+	+	+	+	+	+	+	−	−	−	−	−	−	−	−
aesculin	+	+	+	+	+	+	+	+	−	−	−	−	−	+	−	−
SDS	+	−	−	+	−	−	−	−	−	+	+	+	+	−	+	+
Gas from d-glucose	+	+	−	+	+	+	+	−	+	+	+	+	+	+	−	−
Voges-Proskauer	+	+	−	+	−	−	−	−	−	−	+	+	+	−	+	+
ADH (Moellers’)	+	+	−	−	+	+	+	−	+	+	+	+	+	+	+	+
LDC (Moellers’)	+	+	−	+	+	−	−	−	−	+	−	+	+	+	+	−
ODC (Moellers’)	−	−	−	+	−	−	−	−	−	−	−	−	−	−	−	−
Acid from l-arabinose	−	+	+	−	+	−	+	+	−	−	−	−	−	+	−	−
salicin	+	+	+	+	+	+	+	+	−	−	−	−	−	−	−	−
d-cellobiose	−	−	+	+	+	−	+	+	+	+	−	+	−	+	−	−
l-fucose	−	+	−	−	−	−	−	−	−	−	−	−	−	−	−	−
l-rhamnose	−	−	−	−	−	+	−	−	−	−	−	−	−	+	−	−
Use of dl-Lactate	+	+	+	−	−	−	−	+	−	+	−	−	−	−	+	−
Urocanic acid	+	−	+	−	+	+	−	+	−	+	+	−	−	−	−	−

+, all strains are positive; −, all strains are negative

a Strains included in the cluster are: *A. hydrophila* subsp. *dhakensis* CECT 5744^T^, CECT 5743 and CECT 5745; isolates ABF132, ABF144, ABF145, MA17, MA26, MA131, 133.341, and 133.343; strain CECT4588; and *A. aquariorum* MDC47^T^, MDC310, and MDC317.

**Table 2:** Antimicrobial resistance in the Spanish *A. hydrophila* subsp. *dhakensis* isolates

		MIC (μg mL^−1^)			
Antibiotic	Range	For the 50% tested strains	For the 90% tested strains	Break-points (μg mL^−1^)	Resistant strains (%)
Amoxicillin/Clavulanic (AMC)	128–512	128	256	32^a^	100^b^
Cefoxitin (FOX)	32–128	64	128	32^a^	100^b^
Imipenem (IPM)	0.1–128	32	64	16^a^	75^b^
Ticarcillin (TIC)	128–512	128	256	128^a^	100^b^
Piperacillin (PRL)	0.1–256	0.5	256	128^a^	50^b^
Flumequine (UB)	0.1–64	0.3	64	8^a^	50^b^
Nalidixic acid (NA)	0.1–512	0.5	512	32^a^	37.5^b^
Oxolinic acid (OA)	0.2–64	0.2	32	4^a^	37.5^b^
Erythromycin (ERY)	0.1–64	16	64	8^a^	62.5^b^
Rifampicin (RD)	0.1–4	0.1	2	4^a^	12.5

For cefotaxime (CTX), ceftazidime (CAZ), cefepime (CPM), aztreonan (ATM), kanamycin (K), tetracyline (TET), ciprofloxacin (CIP), levofloxacin (LEF), norfloxacin (NOR), choramphenicol (CHL) and netilmicin (NET), the MIC at which 90% of isolates were inhibited was inferior to 0.5 μg mL^−1^, so all strains were sensitive to these drugs.

a This value of MIC is the cut-off to consider resistance to the antimicrobial (9).

b The two clinical isolates (*i.e.*, 133.341 and 133.343) were resistant.
